# Trunk Posture during Manual Materials Handling of Beer Kegs

**DOI:** 10.3390/ijerph18147380

**Published:** 2021-07-10

**Authors:** Colleen Brents, Molly Hischke, Raoul Reiser, John Rosecrance

**Affiliations:** 1 Department of Environmental and Radiological Health Sciences, College of Veterinary Medicine and Biomedical Sciences, Colorado State University, Fort Collins, CO 80523, USA; Molly.Hischke@colostate.edu (M.H.); John.Rosecrance@colostate.edu (J.R.); 2 Department of Health and Exercise Science, College of Health and Human Sciences, Colorado State University, Fort Collins, CO 80523, USA; Raoul.Reiser@colostate.edu; 3 School of Biomedical Engineering, College of Engineering, Colorado State University, Fort Collins, CO 80523, USA

**Keywords:** manual materials handling, craft brewery, ergonomics, low back injury

## Abstract

Craft brewing is a rapidly growing industry in the U.S. Most craft breweries are small businesses with few resources for robotic or other mechanical-assisted equipment, requiring work to be performed manually by employees. Craft brewery workers frequently handle stainless steel half-barrel kegs, which weigh between 13.5 kg (29.7 lbs.) empty and 72.8 kg (161.5 lbs.) full. Moving kegs may be associated with low back pain and even injury. In the present study, researchers performed a quantitative assessment of trunk postures using an inertial measurement unit (IMU)-based kinematic measurement system while workers lifted kegs at a craft brewery. Results of this field-based study indicated that during keg handling, craft brewery workers exhibited awkward and non-neutral trunk postures. Based on the results of the posture data, design recommendations were identified to reduce the hazardous exposure for musculoskeletal disorders among craft brewery workers.

## 1. Introduction

Back injuries account for more than a third of reported occupational musculoskeletal disorders (MSDs) in the U.S. labor force [1]. Over 50 percent of workers who reported back injuries specified their low back [2]. The overall economic impact of occupational low back pain (LBP) in the U.S. exceeds $50 billion annually [3,4,5,6]. This value includes both direct costs (lost wages and medical treatment) and indirect costs (losses in productivity). During manual material handling (MMH) tasks, workers may be exposed to physical risk factors associated with LBP including awkward or non-neutral postures of the worker’s trunk, long periods of work activity involving the low back, excessive weight of loads moved, and high frequency of lifts or MMH tasks [7,8,9,10,11,12,13,14,15,16,17,18,19]. Manual materials handling tasks and their associated risks for LBP are common in many industries and small businesses including craft breweries [20,21,22].

In the last decade, the number of craft breweries in the U.S. has grown 300% with over 7000 operations in 2020 [23]. The definition of a craft brewery is a brewery that produces less than six million barrels per year [24,25]. Although individual craft breweries typically have small production scales, collectively, the U.S. craft brewing industry accounted for 13% of beer production and for 25% of all sales in the $111.4 billion U.S. beer market [23]. The success and subsequent expansion of the craft brewing industry has created a growing, yet understudied, working population.

Craft breweries typically begin as small businesses that lack the resources for initial investments in mechanized or robotic equipment. The Occupational Health and Safety Administration (OSHA) defines a small business as a company with fewer than 250 employees at a single facility and less than 500 employees overall [26]. As a result, most work in craft breweries is performed through physical labor, even during periods of growth and increased production demands. The most strenuous physical labor in craft breweries involves MMH, which may increase the risk of occupational low back injuries [11]. In contrast to craft breweries, large breweries are almost entirely automated and use robotics for keg handling.

Craft brewery workers frequently handle stainless steel kegs, which are used to store and transport beer. These durable, portable, reusable, pressurized containers have a single spear valve, which is the only opening used to clean, fill, and to drain liquid. Kegs are cleaned and sterilized before they are filled with beer. One of the most popular sizes of kegs used in craft breweries is the half-barrel keg. A half-barrel keg is 59.0 cm high, has a diameter of 41.0 cm, holds 58.7 L (15.5 U.S. gallons), and weighs between 13.5 kg (29.7 lbs.) empty and 72.8 kg (161.5 lbs.) full of beer. Craft brewery workers typically handle full, empty, and partially filled kegs throughout their shift. Given that kegs are relatively heavy objects that must be handled frequently within a craft brewery, it is likely that workers are exposed to physical risk factors associated with LBP and MSDs.

To date, aside from a National Institute for Occupational Safety and Health (NIOSH) Health Hazard Evaluation on brewery delivery workers [20], there have been no published studies assessing occupational risk associated with keg handling. In the U.S., occupational injury data relevant to the brewing industry is categorized by the North American Industry Classification System (NAICS) as ‘breweries’ or the code 31,212 [27]. While the basic process of brewing beer is constant, the size of production leads to different occupational exposures between workers at craft and large breweries. For example, workers at a large brewery that produces 10 million barrels with an automated kegging line will have different exposures to physical occupational risk factors for MSDs compared to workers at a craft (smaller) brewery that produces 10,000 barrels with a manual kegging line. The available occupational injury data associated with beer brewing does not include the size of the brewery. Thus, an analysis of NAICS injury data within the U.S. brewing industry is not specific to brewery size and may lead to ineffective intervention strategies aimed at reducing work-related injuries for large and craft breweries. In order to maximize the positive impact of interventions at craft breweries, it is critical that researchers understand the occupational exposure hazards within craft brewing operations.

The focus of the present study was to perform a quantitative assessment of trunk postures exhibited by workers lifting kegs during operational hours at a craft brewery with a validated inertial measurement unit (IMU)-based kinematic measurement system. Researchers hypothesized that craft brewery workers operating the kegging line are exposed to awkward trunk postures, which have been associated with low back injury. Based on the results of the quantitative assessment, design recommendations to reduce the hazardous exposures were also identified.

## 2. Materials and Methods

### 2.1. Subjects

All five employees who regularly operated the kegging line at a single craft brewery participated in the present study. Each participant provided university approved written informed consent prior to beginning the study. Approval for the study was also obtained from the craft brewery management team prior to beginning the study. Participants were healthy and free of pain or injury at the time of data collection. All study procedures were approved by the Institutional Review Board on Human Subjects at the researchers’ academic institution.

### 2.2. Work Environment

**Figure 1:**
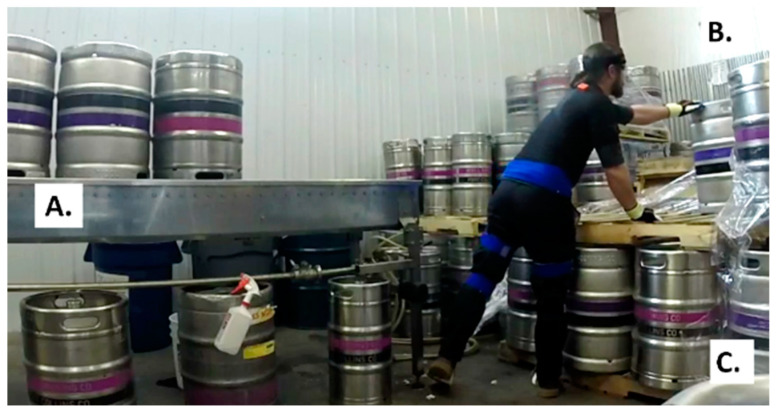
The kegging line work area included the roller conveyor (**A**) and empty kegs on pallets identified as high kegs (**B**) and low kegs (**C**).

The brewery that participated in the present study was the third largest craft brewery in Colorado and 22nd largest craft brewery in the U.S. [24]. In 2016, they brewed over 120,000 barrels with a semi-automatic kegging line [24]. At peak efficiency, the kegging line throughput (clean and fill) varied from 144 to 160 half-barrel kegs per hour [28]. Craft brewery workers manually loaded empty kegs onto an inline roller conveyor that moved the kegs through the cleaning and filling processes. Kegs were cleaned externally, sterilized internally, and then filled with beer. During the task of loading kegs onto the roller conveyor, workers stood between the roller conveyor and incoming pallet of empty kegs (Figure 1). Workers would typically lift, rotate, carry (a short distance), and flip empty kegs upside down. From this position, kegs were then injected with a cleaning solution, decanted, rinsed, and filled with beer.

**Figure 2:**
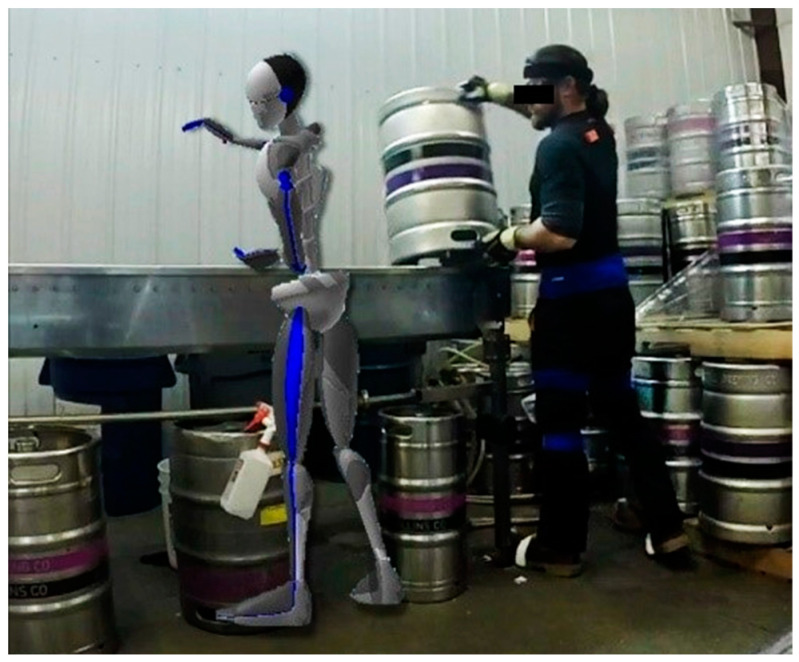
The IMU-based kinematic measurement system generated a 3-dimensional avatar, shown offset from the worker, which corresponded with the craft brewery worker’s motions as they loaded empty kegs onto the kegging line.

Craft brewery workers operating the kegging line were responsible for the flow of the entire line. Workers loaded empty kegs onto the line, monitored the automated cleaning and filling aspects of the line, checked tank pressures to ensure appropriate flow of beer, and cleared space for forklift operators to deliver pallets of empty kegs. During busy shifts, forklift operators needed to work quickly and placed pallets wherever they could find space near the line. Forklift operators typically delivered two pallets of empty kegs stacked on top of each other (eight kegs on the top pallet and eight kegs on the bottom pallet) (Figure 1). Workers transferred empty kegs from the upper pallet to the roller conveyor, removed the top pallet, and then transferred the kegs from the lower pallet to the roller conveyor, and then removed the bottom pallet. For the purposes of the present study, empty half-barrel kegs on the upper pallet were referred to as high kegs with a high keg lift necessary to move the keg. The half-barrel kegs on the lower pallet were identified as low kegs with a low keg lift necessary to move the keg. At this craft brewery, workers manually performed both high and low keg lifts. The vertical origin height of the lift was 70 cm for the low kegs and 140 cm for the high kegs. This value represented the vertical distance between the lower pallet height and distance to the top rim of the keg. The vertical rail height of the roller conveyor was 169 cm from the floor. Craft brewery workers reached across the pallet or walked around it to lift empty kegs. During this MMH task, workers gripped the top rim with one hand (on the same size as the spear valve), tipped the keg so they could grip the bottom rim, and lifted the keg. Workers flipped the keg before placing it onto the roller conveyor (Figure 2). Depending on how much beer is scheduled to be kegged, a craft brewery worker typically handles as many as 600 or as few as 100 kegs over the course of an eight-hour shift. For the present study, craft brewery workers handled 64 empty half-barrel kegs from stacked pallets (32 high lifts and 32 low lifts). Workers handled 64 empty half-barrel kegs within approximately 45 min.

### 2.3. Instrumentation and Procedure

Study participants were fitted with 17 IMU sensors (Xsens MVN MTw Awinda) attached to their upper and lower limbs as well as their trunk. Previous studies have simulated MMH tasks with asymmetric lifting, lowering, pushing, and pulling using IMUs (as individual sensors and as part of a kinematic measurement system). The use of IMUs in this context has been confirmed as an acceptable form of human motion measurement after multiple studies compared IMUs to the gold-standard optical motion capture (OMC) systems and with the lumbar motion monitor (LMM) [29,30,31,32,33].

Workers’ trunk postures were measured as they operated the kegging line. Sensor collection was recorded at 60 Hz. Within each IMU sensor, the gyroscope had a range of +/− 2000 deg/s^2^ with noise of 0.01 deg/s√Hz, the accelerometer had a range of +/− 160 m/s with noise of 200 µg/√Hz, and the magnetometer had a range of +/− 1.9 Gauss with a noise of 0.2 mGauss/√Hz [34]. System calibration procedures were performed in accordance with the manufacturer’s instructions. Workers stood in a position referred to as the ‘n-pose’ (standing straight with hands at side) to calibrate the system (Xsens, Enschede, NT). Data were recorded in MVN Studio BIOMECH software (Xsens, Enschede, the Netherlands) on a laptop computer.

Environmental magnetic interference was tested before data collection began and was monitored during data collection. At no time during data collection did magnetic interference exceed the manufacturer’s acceptable levels of interference. While monitoring environmental magnetic interference, the authors did not observe differences between the final and earlier keg transfers. The combination of updated sensor fusion algorithms (which decreased the reliance on the magnetometer) and the short recording sessions (45 min) effectively minimized any ferromagnetic interference [34,35,36].

For the purposes of this study, the start of a keg lift was defined by when the worker grasped the keg from the pallet and ended when they placed the keg on the roller conveyor. A 3-dimensional kinematic avatar was generated from a proprietary synthesis of sensor data, subject-specific anthropometric measurements, and a biomechanical algorithm as part of the IMU-based kinematic measurement system, allowing visual clarification of a working kinematic system (Figure 2) [34].

### 2.4. Data Processing and Analysis

#### 2.4.1. Craft Brewery Worker Trunk Posture

Keg lift events and the corresponding height (high, low) were identified using video recordings and sensor fusion data for each participant. Video data were time-synced with the sensor fusion data.

Orientation data from the sensors were calculated using a proprietary fusion sensor algorithm, XKF3-hm, by Xsens [34]. The model outputted orientation data as both Euler angles (for visualization) and quaternions (for calculations), which were converted into rotation angles using a custom code generated in MATLAB 2017b (Natick, MA, USA) [34,37].

The proprietary sensor fusion algorithm provides both sensor orientation as well as body segment orientation. To treat the trunk as a rigid body researchers extrapolated orientation information from the posterior pelvic and anterior sternum (T8) sensors to estimate trunk posture angles. Using a custom R code in RStudio Version 1.0.136, 2016 (Boston, MA, USA), keg lift events and angular displacement values were combined to characterize the worker’s trunk posture while they operated the kegging line. Positive angular displacement values corresponded to worker’s trunk flexion in the sagittal plane, left axial rotation, and right lateral flexion.

#### 2.4.2. Estimating Risk of Low Back Pain Using the Lifting Fatigue Failure Tool (LiFFT)

Researchers applied the Lifting Fatigue Failure Tool (LiFFT) to estimate the risk of low back disorder development among craft brewery workers as they handled kegs. This validated tool applies the fatigue failure process to predict risk of a low back outcome by estimating cumulative damage for jobs that require manual materials’ handling and lifting tasks [38,39]. Based on the daily dose and cumulative damage, jobs were categorized as high, medium, or low risk for a low back disorder outcome.

#### 2.4.3. Statistical Analysis

Descriptive statistics (mean, standard deviation) were conducted for each posture exposure metric across all participants. Trunk posture data were determined to be normally distributed with equal sample sizes. Statistical analysis consisted of a repeated measures design and univariate fixed effects analysis of variance (ANOVA) for each torso angular displacement as a function of lifting height origin. The lift height was the condition (low and high) and the within-subject factor. Subjects were treated as a random factor to account for inherent subject to subject variability or variability due to individual differences. Lifts were averaged across subjects for high and low origin positions separately. The interaction between subject and lift condition (independent variables) was investigated. The resulting trunk postures (in sagittal, axial, and transverse planes) were the outcomes of interest (dependent variable). Post-hoc analysis consisted of least square means to examine significant effects. Statistical significance was determined at *p* < 0.05. Statistical analysis was performed using RStudio Version 3.5.3, 2019 (Boston, MA, USA).

## 3. Results

**Figure 3:**
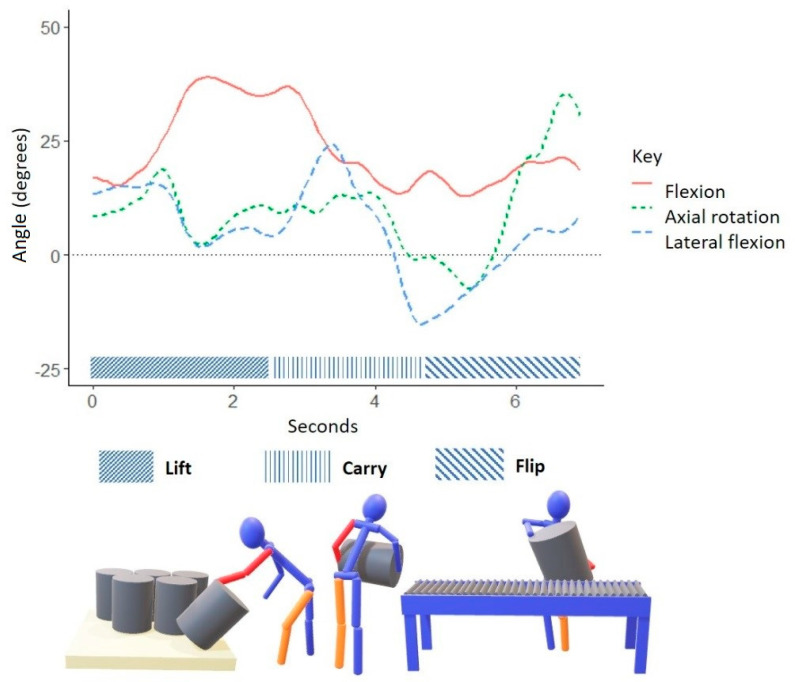
Graphic representation of typical trunk postures exhibited by a craft brewery worker conducting low keg lifts. A solid red line represents trunk flexion and extension in the sagittal plane. A short-dashed green line represents trunk axial rotation in the transverse plane. A long-dashed blue line represents lateral trunk flexion in the frontal plane.

**Figure 4:**
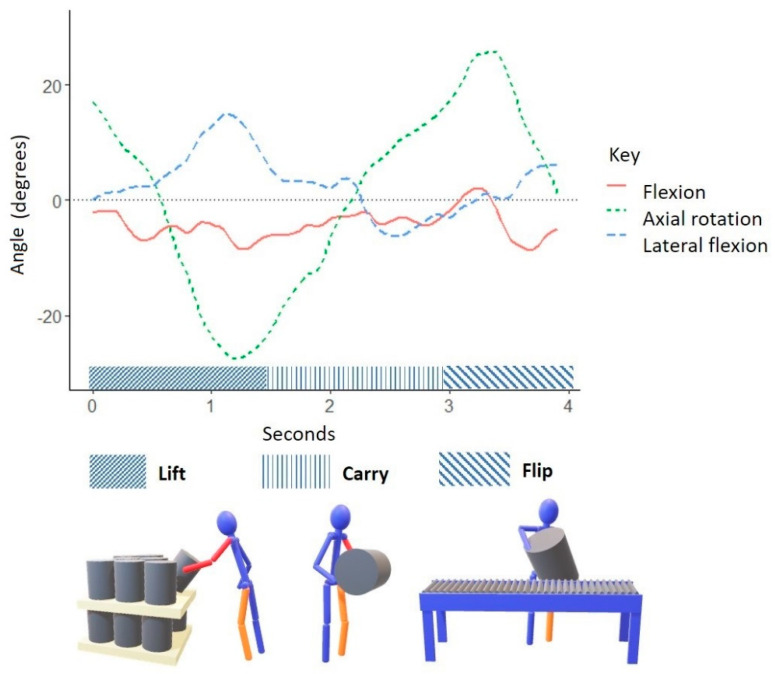
Graphic representation of typical trunk postures exhibited by a craft brewery worker conducting high keg lifts. A solid red line represents trunk flexion and extension in the sagittal plane. A short-dashed green line represents trunk axial rotation in the transverse plane. A long-dashed blue line represents lateral trunk flexion in the frontal plane.

The proprietary sensor fusion algorithm for the IMU-based kinematic measurement system incorporated the participants’ anthropometric variables (shown in Table 1) when determining trunk kinematics. During a typical low keg lift, the worker’s trunk posture began in forward flexion and gradually returned to an upright position while simultaneously moving from right to left lateral flexion and trunk axial rotation to the left before placing the keg on the roller conveyor (Figure 3). When lifting high kegs, the worker began with right axial trunk rotation and left lateral trunk flexion, then gradually transitioned to left axial rotation with right lateral trunk flexion and slight trunk extension as they placed the keg on the roller conveyor (Figure 4).

Table 2 displays the magnitudes of the workers’ trunk postures and direction while they operated the kegging line. Results of the fixed effect ANOVA are shown in Table 3. For the lift height condition main effect, there was sufficient evidence of a relationship among several variables (*p* > 0.05). There was sufficient evidence to suggest an interaction between subject and lift height condition among several variables. Overall, during low keg lifts, workers exhibited larger magnitudes of the following trunk postures: right, left, and mean lateral trunk flexion; right axial rotation, mean flexion, maximum flexion, and maximum extension. During high keg lifts, workers exhibited greater magnitudes of mean and left axial rotation.

Trunk posture was assessed for each lift repetition among the two keg lift heights for all five worker participants. The height of the lift was the main effect of interest. Looking strictly at the ANOVA of the main effect, the researchers had sufficient evidence of a relationship between trunk posture and lift height for the following trunk posture variables: right and left lateral flexion, right and mean axial rotation, and trunk flexion (*p* < 0.05). The interaction effect of height and subject was statistically significant for all trunk posture variables except mean axial rotation, right axial rotation, and minimum flexion (*p* < 0.05). Results of the fixed effects ANOVA suggested that while there was evidence of a relationship between lift height and a worker’s trunk posture, individual differences among workers also contributed to the observed values. For both lift heights, workers exhibited greater magnitudes of left axial rotation (compared to right) and greater magnitudes of right lateral flexion (compared to left). Overall, workers would rotate to the left and lean to the right when handling empty kegs.

Researchers applied the LiFFT cumulative damage measure to estimate the risk of a worker developing a low back disorder from operating the kegging line. The risk model required the maximum horizontal distance between the lifted load and the workers’ center of mass, weight of the load, and the number of repetitions during an eight-hour shift [38]. Craft brewery workers often carried the empty keg close to their body (seen in Figure 2, Figure 3 and Figure 4), but extended their reach when reaching for the keg as well as flipping and placing the keg onto the conveyor roller. The worker’s posture of forward flexion plus reaching or pushing the keg represented the greatest peak horizontal distances during keg handling. Researchers applied the mean peak horizontal distance (63.2 cm, 24.9 in) and assumed kegs were empty (13.5 kg, 29.7 lbs.) to calculate a peak moment arm 81.3 Nm (60 ft lbs.). As described earlier, kegging demands varied from 100 to 600 kegs per eight-hour shift. The present study observed 64 lifts, which equated to 324 lifts per shift. Thus, researchers applied LiFFT to all three lifting frequency scenarios. Risk estimate was calculated assuming kegs were empty for all three lifting scenarios. Table 4 provides the LiFFT assessment of risk associated with keg handling task characteristics. In terms of cumulative damage and percent risk, handling empty kegs under three common frequency scenarios equated to a medium risk or 30–50% of total damage. For example, according to LiFFT, a worker who lifts an empty keg 100 times with a peak moment of 81.3 Nm (60 ft lbs.) will accrue a cumulative damage daily dose of 0.0033, a figure associated with an estimated risk of 30.6% of experiencing a low back disorder (i.e., medium risk).

## 4. Discussion

### 4.1. Trunk Postures

Craft brewery workers experienced less trunk flexion during high keg lifts compared to low keg lifts. Given that empty kegs typically arrived stacked, the vertical distance a worker had to lift a low keg from the pallet to conveyor roller was 70.4% greater than the distance the worker had to lift a high keg. This task configuration likely contributed to differences in measured trunk flexion among workers in the present study.

While there was sufficient evidence of a relationship between maximum trunk flexion and lift height (with workers experiencing greater trunk flexion during low lifts), the interaction between lift height and worker was also significant (*p* < 0.05). Individual worker lift strategies likely contributed to differences between trunk flexion and lift characteristic. During data collection, workers were observed reaching across pallets to grasp the high keg top rim compared to walking around the pallet to access low kegs. This difference in keg grasping by different workers may have contributed to observed increases in worker trunk flexion. The limited sample size could have allowed the workers’ unique lifting styles to have a greater influence on the results.

Workers exhibited greater lateral trunk flexion during low keg lifts compared to high keg lifts. Workers leaned, or laterally flexed, 34% farther to the right during low keg lifts. Comparing lateral trunk flexion direction, workers exhibited over 80% greater magnitudes of lateral trunk flexion during low lifts compared to high lifts (88% greater lateral flexion to the right during high lifts and 78% greater lateral flexion to the left during low lifts). The workers’ trunk axial rotations did not vary significantly between high and low keg lifts. Workers exhibited 120% greater trunk axial rotation to the left compared to the right during keg handling (given that the mean trunk rotation was five degrees for low lifts and 20 degrees for high lifts).

Overall, workers exhibited greater trunk flexion and greater lateral trunk flexion when lifting low kegs compared to high kegs. Regardless of keg lift height, workers exhibited greater lateral trunk flexion to the right and greater axial trunk rotation to the left. The current kegging line design was such that incoming empty kegs arrived alongside the roller conveyor. If a worker stood between the roller conveyor and pallet of empty kegs, they could lean to the right, grasp the keg’s top rim, lift, rotate, and place the inverted keg on the roller conveyor located to their left (as shown in Figure 1 and Figure 2).

### 4.2. Estimates of Cumulative Damage Derived from LiFFT Risk Assessment Tool

Under empty keg assumptions across multiple task repetition scenarios, researchers estimated workers to be at a ‘medium risk’ for developing a low back disorder. However, kegs may be returned to the craft brewery with residual amounts of beer. Kegs are opaque; thus, workers do not know the true weight of the keg until they initiate the lift. Researchers also applied the LiFFT model to estimate the risk of developing a low back disorder if a craft brewery worker encountered an entire kegging shift of kegs with a quarter of residual beer in each (28.58 kg, 63 lbs.) using the same postures for assumed empty kegs. If a worker lifted 100 non-empty kegs, using LiFFT, it was estimated that the craft brewery worker could experience a peak moment of 171 Nm (126 ft. lbs.) and accrue a cumulative damage daily dose of 0.0962, a figure associated with an estimated risk of 66.4% of experiencing a low back disorder (i.e., high risk).

While it is unlikely that workers would experience an entire shift of partially full kegs as they operated the kegging line, workers described finding (and researchers observed) sporadic non-empty kegs during kegging operations. When workers encounter non-empty kegs, they must manually decant the kegs because the kegging line is not designed to drain kegs before cleaning. Not only does the presence of non-empty kegs disrupt workflow, non-empty kegs also increase the workers’ estimated risk of a low back disorder. Table 5 presents a hypothetical scenario where, on a slow kegging shift (100 kegs), a worker handles a combination of empty and partially full kegs. According to LiFFT analysis, that kegging task exposes the worker to a high risk of developing a low back disorder, with a total cumulative damage of 0.1633 and an estimated probability of a high risk job of 71.4%.

### 4.3. Comparisons to Other Occupational Groups

Although there are no other published studies to date that quantify craft brewery worker trunk posture during keg handling, previous studies have characterized occupational physical exposures associated with MMH tasks in other industries. Previous studies used wearable motion capture devices to investigate physical risk exposures associated with LBP among reforestation workers, grocery stockers, nurses, and distribution workers conducting MMH tasks [40,41,42,43]. Common occupational exposures to physical risk factors for LBP between craft brewery workers and the comparison occupational groups included awkward and non-neutral postures while handling heavy loads.

The maximum trunk flexion measured among craft brewery workers was less than that reported for distribution workers [42], reforestation workers [41], and nurses [40]. However, maximum trunk flexion measured among craft brewery workers during high lifts was greater than that reported for grocery workers [43]. The maximum lateral trunk flexion measured among craft brewery workers during high lifts was below that reported for warehouse distribution workers [42]. However, maximum lateral trunk flexion measured among craft brewery workers was greater than that reported for reforestation workers [41]. Craft brewery workers operating the kegging line exhibited greater trunk axial rotation than grocery workers unloading carts [43]. The comparisons between the present craft brewery study and other occupational groups are outlined in Table 6.

Differences in the work environment and task nature likely contributed to the postural differences between workers in craft breweries and other industries. Studies quantifying posture among distribution workers [42] and nurses [40] measured workers throughout their entire shift whereas craft brewery workers, grocery workers [43], and reforestation workers [41] were measured during specific tasks within a single shift. Tree saplings, as an example of task differences, are typically shorter than half-barrel kegs (stacked or single). The difference in lifted item height (saplings vs. kegs) requires reforestation workers to exhibit greater magnitudes of trunk flexion as they complete planting tasks compared to craft brewery workers handling kegs [41]. Marras et al. (2010) assessed worker posture throughout the work shift (from forklift driving to building and breaking down pallets) among workers in grocery, automotive, merchandise, and clothing distribution facilities. Nurses studied by Schall et al. (2016) handled patients, charts, and monitored equipment. Grocery workers handled beverage cases (bottles and cans), among other stock, from a cart [43]. Craft brewery workers also often handled packaged beer in cases of cans and bottles; however, the present study focused on keg handling tasks.

Demographics of the workforce might contribute to postural differences between occupational groups. Craft brewery workers were older than reforestation workers [41], younger than distribution workers [42], and similar in age to grocery workers [43] and nurses [40]. A worker’s age may increase their risk of developing LBP, especially during manual materials’ handling tasks [44]. The present study only measured trunk postures exhibited by male craft brewery workers. Likewise, workers studied in reforestation [41], grocery [43], and distribution [42] industries were predominately, if not entirely, male (see Table 6 for comparisons). By studying trunk kinematics of only male workers, the present study has limited generalizability as it excludes female workers. As the craft brewing industry continues to expand, the population of craft brewery workers will grow in size and diversity. To ensure a safe and healthy craft brewing workforce, it is critical that researchers and practitioners understand task demands associated with MMH in this understudied yet growing industry.

Other researchers, from the studies cited above, characterized worker posture and movement using various wearable motion capture devices. The lumbar motion monitor (LMM), a wearable exoskeleton device, measured postures exhibited among distribution center workers [42] and grocery workers [43]. Inertial measurement unit sensors measured postures exhibited among reforestation workers [41], nurses [40], and craft brewery workers. The researchers of the present study applied an IMU system with 17 sensors to quantify worker posture compared to other studies that used four sensors [41] and three sensors [40] placed on key body regions. Systems with fewer IMU sensors may be less expensive with a quicker setup, but may also generate simplified posture data. For example, the three-sensor system used by Schall et al. (2016) only reported trunk sagittal flexion. Wearable motion capture devices are tools to quantify human trunk posture, and the scope of the research question should drive technology tool selection.

### 4.4. Design Recommendations

As part of the present study, the researchers presented the findings on trunk postures exhibited by workers as they handled kegs to the packaging department at the craft brewery where the research was conducted. Researchers discussed the findings with the packaging department and collaborated on developing design modifications to reduce workers’ exposure to risk factors for LBP and MSDs. Specifically, the recommendations targeted the initial lift, carry, and flip stages of keg handling.

#### 4.4.1. Recommendation 1: Empty Kegs at One Level

In the current kegging line, kegs arrived at two levels (high and low heights). As a result, workers exhibited different trunk postures as they handled high or low kegs. Postures exhibited during both keg height conditions were considered to be awkward or non-neutral. Craft brewery workers were at an increased risk of developing LBP during both height conditions. The researchers recommend modifying the workstation so that empty kegs are delivered at a single optimal height. Incoming empty kegs could be loaded at one height, then a sloped roller conveyor gradually delivers the kegs to the cleaning and filling machinery. If kegs arrive at the optimal height (ideally the same height as the destination), the worker would simply need to rotate the keg. This approach would require kegs to be delivered as single pallets, or onto a sloped conveyor so that kegs eventually reached this optimal height before being flipped.

#### 4.4.2. Recommendation 2: Redesign Workstation Layout

Forklift operators were often rushed and hastily placed pallets at irregular locations in the work area which required workers on the kegging line to carry empty kegs various distances to the roller conveyor. If workers carried kegs a consistent and short distance, they could have more time to focus on managing the line or additional brewing tasks. The researchers recommend three possible solutions to improve the carry phrase of keg handling: floor markings, a safety mirror, and/or a platform with casters to move pallets of kegs to their optimal location. The addition of floor markings could standardize where forklift operators place incoming pallets. During busy shifts, if a forklift driver has to deposit pallets outside the floor markings, the pallets could be on casters so that the worker could easily reposition the pallet before handling the kegs. Installing a safety mirror could help forklift drivers visually identify where to place pallets of kegs. Forklift operators could also use the safety mirror to see the worker operating the kegging line, even when they are blocked behind existing stacks of kegs. If a forklift operator does not see (or know that) a kegging line worker is behind stacks of existing kegs, there is a risk that the forklift operator could push pallets of new kegs into the worker. Upon completion of the present study, the craft brewery installed a safety mirror above the kegging line.

#### 4.4.3. Recommendation 3: Mechanical Keg Handling and Robotics

The kegging line requires kegs to be placed upside down, but empty kegs typically arrive upright. While an upright position allows the consumer to access the keg’s spear valve, kegs need to be upside down to be cleaned and filled with fresh beer. Therefore, under current task configurations, workers flip the kegs as they place them on the roller conveyor.

Craft breweries with resources to invest in mechanized equipment or alternative practices (often larger establishments) could automate kegging operations so that any combination of the lift, carry, and flip stages of keg handling may be eliminated.

Specialized vacuum lift-assist devices, often used by craft brewery workers to place full kegs on pallets, could be applied to empty keg handling. To use a vacuum lift-assist device, the worker simply activates the pump, guides the lifted item from the origin to the destination, and releases the vacuum. Some vacuum lift-assist devices have rotational capabilities, which may be used to reorient empty kegs. Vacuum lift-assist devices eliminate the weight of the load, but still require a worker to maneuver the object.

A robotic keg de-palletizer could eliminate all manual keg handling among workers. The craft brewery that participated in the present study currently handles full kegs robotically. After empty kegs are cleaned and filled with beer, a robotic palletizer arranges the full kegs on a pallet. Forklift operators then transport these pallets across the craft brewery. The manual handling of empty kegs could be eliminated by applying a similar robotic keg-handling technology. However, automation is not always economically feasible for many small developing craft breweries that lack resources to invest in sophisticated materials handling equipment.

#### 4.4.4. Recommendation 4: Outsourcing and Collaborating with Contract Breweries

Craft breweries may outsource empty keg handling by contracting with a third party to reposition empty kegs with the spear valve pointed downwards. A forklift operator would then load the pallet of correctly positioned kegs onto the kegging line. While this technique eliminates manual keg handling among craft brewery workers, the MMH requirements have simply been shifted to a separate workforce.

Instead of modifying their own brewing facilities, craft breweries could hire contract breweries to reduce exposures to physical risk factors associated with keg handling. Contract breweries are brewing facilities whose primary purpose is to provide an environment for small craft breweries to brew beer [24]. In contrast to typical craft breweries, contract breweries do not have to rationalize equipment costs with investments toward personal branding, tap room management, and other related business demands. Historically, controversy has surrounded contract breweries as the operation itself depersonalizes the brewing process by ‘producing local beer somewhere else.’ However, the benefits of accessing large scale production equipment may outweigh the controversy surrounding contract breweries. Some craft breweries utilize contract breweries as an intermediate step to continue their growth as they gather resources to expand their own facility, while others integrate contract brewing into their permanent production strategies. Today, both single craft breweries and collections of multiple craft breweries regularly utilize contract brewing as a means of producing more beer than what is feasible at their own facilities.

### 4.5. Limitations and Suggestions for Further Research

In the present study, trunk postures exhibited by workers applied directly to the kegging line operation in a single craft brewery were observed. While the sample size was small (five participants), it successfully captured all of the workers conducting that task at the craft brewery. This craft brewery is the second largest in the county and the largest operation that handles empty kegs using both manual (for empty kegs) and automatic (for filling and handling full kegs) techniques for keg handling. Keg handling procedures, and subsequent workers’ postures, may vary across craft breweries throughout Colorado.

The tool utilized to measure worker motions recorded comprehensive information about the entire body and velocity, acceleration in addition to position and angular displacement. While the focus of the present study was strictly angular displacement of the trunk, future analysis could investigate other body parts such as the shoulder and knee.

This study only investigated awkward or non-neutral postures and heavy loads (assumed to be empty half-barrel kegs) as occupational physical risk factors affecting craft brewery workers. While researchers focused on individual component rotations, future studies could combine rotations about multiple anatomical axes to represent more complex multiplanar postures and movements. In addition to postures, other occupational physical risk factors associated with MMH and LBP include long periods of work activity, excessive weight of loads moved, and high frequency of lifts [7,8,12,13,14,15]. Workers operating the kegging line were also responsible for additional jobs throughout the brewery, thus assessing posture during one task may not represent their entire work-demands. Workers exhibited non-neutral trunk postures as they lifted pallets from the floor, crouched under pipes, handled large bags of ingredients, and conducted other common brewing tasks. The present study investigated keg handling characteristics within a single craft brewery, but operations vary among craft breweries depending on the production scale and business scope. The researchers recommend that future studies investigate worker motions throughout multiple craft brewing tasks and across different craft brewing facilities.

## 5. Conclusions

Craft brewery workers are exposed to awkward postures and heavy loads as they handle kegs, and are at an increased risk for developing MSDs and LBP. Researchers estimated that workers were at a ‘medium risk’ of developing a low back disorder if they only handled truly empty kegs. If workers handled partially filled kegs, the risk increased to ‘high risk’. Researchers outlined design recommendations to reduce the awkward postures and heavy lifting associated with operating the kegging line.

The U.S. craft brewing industry is rapidly growing with workers who are exposed to physical risk factors associated with MSDs. Despite this industry growth, little is understood about work-demands among craft brewery workers. Thus, it is worthwhile for researchers to continue research among craft brewery workers to characterize and assess work-demands beyond keg handling. As researchers and practitioners better understand exposures to physical risk factors during craft brewing work, they can apply that knowledge to developing effective interventions to reduce the risk of MSDs and to improve the quality of work life among craft brewery workers.

## Figures and Tables

**Table 1:** Demographic and anthropometric data of the study participants (*n* = 5).

Variable	Units	Subject 1	Subject 2	Subject 3	Subject 4	Subject 5
Age	years	40	29	30	28	29
Body mass	kg	88.9	78.8	74.9	80.7	80.9
Body height	cm	181.0	177.5	183.5	177.0	173.0
Foot size	cm	31.0	33.0	31.3	32.5	30.3
Arm span	cm	178.0	176.0	175.0	175.5	172.5
Ankle height	cm	9.5	9.5	10.0	10.5	11.5
Hip height	cm	99.5	91.5	99.0	97.0	90.0
Hip width	cm	28.5	30.5	27.0	27.5	26.0
Knee height	cm	54.5	47.5	52.0	52.5	50.0
Shoulder width	cm	44.5	40.0	38.0	37.2	27.0
Shoe sole height	cm	4.3	3.5	4.5	4.0	4.8

**Table 2:** Craft brewery trunk posture. Mean angle (SD). Significant values (*p* ≤ 0.05) have *.

Motion	High (◦)	Low (◦)	*p*-Value
Mean lateral flexion	3.74 (5.63)	4.59 (7.02)	0.19
Left lateral flexion	−5.30 (7.63)	−8.31 (8.46)	0.31
Right lateral flexion	13.47 (7.53)	19.01 (9.08)	0.19
Mean axial rotation	8.10 (7.59)	6.44 (8.28)	0.04 *
Left axial rotation	21.30 (11.17)	20.33 (11.76)	0.16
Right axial rotation	−4.98 (8.14)	−6.84 (8.71)	0.79
Mean flexion	16.13 (8.06)	19.50 (10.55)	0.02 *
Maximum flexion	23.80 (10.13)	30.89 (13.38)	0.01 *
Minimum flexion	8.86 (8.22)	9.92 (9.04)	0.08

**Table 3:** Summary of the F-statistic (*p*-value) for the fixed effect analysis of variance (ANOVA) test for worker, lift height, and the interaction between worker and lift height (df = degrees of freedom).

Motion	Worker (df = 4)	Height (df = 1)	Worker × Height (df = 4)
Mean lateral flexion	33.56 (0.001)	1.17 (0.28)	31.69 (0.001)
Left lateral flexion	44.31 (0.001)	25.86 (0.001)	13.05 (0.001)
Right lateral flexion	36.95 (0.001)	62.40 (0.001)	38.90 (0.001)
Mean axial rotation	65.57 (0.001)	7.93 (0.005)	0.78 (0.54)
Left axial rotation	54.59 (0.001)	1.62 (0.20)	4.01 (0.003)
Right axial rotation	28.69 (0.001)	5.74 (0.02)	2.18 (0.07)
Mean flexion	122.88 (0.001)	45.31 (0.001)	8.33 (0.001)
Maximum flexion	91.82 (0.001)	87.79 (0.001)	16.60 (0.001)
Minimum flexion	95.63 (0.001)	8.89 (0.003)	1.20 (0.31)

**Table 4:** Estimates of cumulative damage derived from the LiFFT risk assessment tool for empty keg (13.5 kg, 29.7 lbs.) handling.

Peak Moment (Nm, ft. lbs.)	Repetitions per Workday	Cumulative Damage	Low Back Risk/Estimated Injury Risk (%)
81.3, 60	324 (observed)	0.0112	43.2 (medium risk)
81.3, 60	100 (minimum)	0.0033	30.6 (medium risk)
81.3, 60	600 (maximum)	0.0196	49.4 (medium risk)

**Table 5:** Estimates of cumulative damage derived from the LiFFT risk assessment tool for various keg weights.

Load	Peak Moment (Nm, ft. lbs.)	Load (kg, lbs.)	Repetitions per Workday
Empty keg	81.3, 60	13.6, 30	85
Quarter full keg	170.8, 126	28.6, 63	10
Half full keg	260.3, 192	43.5, 96	5

**Table 6:** Comparisons to other occupational groups. SD represents standard deviation. Three to four workers were measured per task, with 306 subjects overall.

Industry	Authors	Sample Size	% Male	Mean Age (SD)	% Shift	Measurement Device	Peak Flexion (◦)
Craft Brewery	Present study	5	100	31.0 (4.5)	20	17 IMUs	23.8, high keg lift 30.89, low keg lift
Distribution	Marras et al., 2010	4	83	33.9 (10.7)	50	LMM	51.5
Grocery	Davis et al., 2014	15	100	31.0 (7.7)	100	LMM	29.0
Nursing	Schall et al., 2016	36	0	30.8 (10.1)	100	3 IMUs	35.9
Reforestation	Granzow et al., 2018	14	100	26.9 (6.0)	100	4 IMUs	75.2

## Data Availability

Not applicable.

## References

[1] Bureau of Labor Statistics U.S. (2018). Department of Labor. The Economics Daily: Back Injuries Prominent in Work-Related Musculoskeletal Disorder Cases in 2016.

[2] Bureau of Labor Statistics U.S. Department of Labor (2018). Table 2. Number, Incidence Rate, and Median Days Away from Work for Nonfatal Occupational Injuries and Illnesses Involving Days Away from Work for Musculoskeletal Disorders by Any Part of Body and Ownership, National, 2016.

[3] Dagenais S., Caro J., Haldeman S. (2008). A systematic review of low back pain cost of illness studies in the United States and internationally. Spine J..

[4] Odole A., Akinpelu A., Adekanla B., Obisanya O. (2011). Economic Burden of Low Back Pain on Patients Seen at the Outpatient Physiotherapy Clinics of Secondary and Tertiary Health Institutions in Ibadan. J. Niger. Soc. Physiother..

[5] Jones T., Kumar S. (2001). Physical Ergonomics in Low-Back Pain Prevention. J. Occup. Rehabil..

[6] Gatchel R.J., Schultz I.Z. (2014). Handbook of Musculoskeletal Pain and Disability Disorders in the Workplace.

[7] Basahel A.M. (2015). ScienceDirect Investigation of work-related Musculoskeletal Disorders (MSDs) in warehouse workers in Saudi Arabia. Procedia Manuf..

[8] Coenen P., Kingma I., Boot C.R.L., Bongers P.M., van Dieën J.H. (2014). Cumulative mechanical low-back load at work is a determinant of low-back pain. Occup. Environ. Med..

[9] van Dieën J.H., Faber G.S., Loos R.C.C., Paul P., Kuijer F.M., Kingma I., Van Der Molen H.F., Frings-Dresen M.H.W. (2010). Validity of estimates of spinal compression forces obtained from worksite measurements. Ergonomics.

[10] Zurada J., Karwowski W., Marras W. (2004). Classification of jobs with risk of low back disorders by applying data mining techniques. Occup. Ergon..

[11] Waters T., Putz-Anderson V., Garg A., Fine L. (1993). Revised NIOSH equation for the design and evaluation of manual lifting tasks. Ergonomics.

[12] de Looze M.P., Kingma I., Thunnissen W., van Wijk M.J., Toussain H.M. (1994). The evaluation of a practical biomechanical model estimating lumbar moments in occupational activities. Ergonomics.

[13] Potvin J.R. (2008). Occupational spine biomechanics: A journey to the spinal frontier. J. Electromyogr. Kinesiol..

[14] Korkmaz S.V., Hoyle J.A., Knapik G.G., Splittstoesser R.E., Yang G., Trippany D.R., Lahoti P., Sommerich C.M., Lavender S.A., Marras W.S. (2006). Baggage handling in an airplane cargo hold: An ergonomic intervention study. Int. J. Ind. Ergon..

[15] Punnett L., Fine L.J., Keyserling W.M., Herrin G.D., Chaffin D.B. (1991). Back disorders and nonneutral trunk postures of automobile assembly workers. Scand. J. Work. Environ. Health.

[16] Faber G.S., Kingma I., van Dieën J.H. (2007). The effects of ergonomic interventions on low back moments are attenuated by changes in lifting behaviour. Ergonomics.

[17] Lavender S., Marras W., Ferguson S., Splittsteosser R., Yang G. (2012). Developing physical exposure-based back injury risk models applicable to manual handling jobs in distribution centers. J. Occup. Environ. Hyg..

[18] Magora A. (1975). Investigation of the relation between low back pain and occupation. Scand. J. Rehabil. Med..

[19] Marras W.S. (1993). Dynamic Measures of Low Back Performance.

[20] Mcglothlin J.D., Steven C.P.E., Wurzelbacher J. (2000). HHE Report No. HETA 97-0076-2805 Coors Distributing Company Golden, Colorado.

[21] Marras W.S., Lavender S.A., Ferguson S.A., Splittstoesser R.E., Yang G. (2010). Quantitative Dynamic Measures of Physical Exposure Predict Low Back Functional Impairment. Spine (Phila. Pa. 1976).

[22] NORA Manufacturing Sector Council (2018). National Occupational Research Agenda for Manufacturing.

[23] Brewers Association Craft Beer Sales by State. https://www.brewersassociation.org/statistics/by-state/.

[24] Brewers Association National Beer Sales & Production Data. Brewers Association. https://www.brewersassociation.org/statistics/national-beer-sales-production-data/.

[25] Brewers Association Industry Updates: U.S. Bureau of Labor Statistics Data Suggests Improved Brewery Safety—Brewers Association. https://www.brewersassociation.org/industry-updates/u-s-bureau-labor-statistics-data-suggests-improved-brewery-safety/.

[26] Michaels D. (2015). (OSHA) Consultation Policies and Procedures Manual (CSP 02-00-003).

[27] Bureau of Labor Statistics (2016). Nonfatal Occupational Injuries and Illnesses with Days Away from Work 2015.

[28] Lambrechts Konstruktie N.V. (2016). Lambrechts News 2016.

[29] Khurelbaatar T., Kim K., Lee S.K., Kim Y.H. (2015). Consistent accuracy in whole-body joint kinetics during gait using wearable inertial motion sensors and in-shoe pressure sensors. Gait Posture.

[30] Faber G.S., Chang C.C., Dennerlein J.T., van Dieën J.H. (2016). Estimating 3D L5/S1 moments and ground reaction forces during trunk bending using a full-body ambulatory inertial motion capture system. J. Biomech..

[31] Kim S., Nussbaum M.A. (2013). Performance evaluation of a wearable inertial motion capture system for capturing physical exposures during manual material handling tasks. Ergonomics.

[32] Schall M.C., Fethke N.B., Chen H., Gerr F. (2015). A comparison of instrumentation methods to estimate thoracolumbar motion in field-based occupational studies. Appl. Ergon..

[33] Miezal M., Taetz B., Bleser G. (2016). On Inertial Body Tracking in the Presence of Model Calibration Errors. Sensors.

[34] Paulich M., Schepers H.M., Rudigkeit N., Bellusci G. (2018). Xsens MTw: Miniature Wireless Inertial Motion Tracker for Highly Accurate 3D Kinematic Applications.

[35] Roetenberg D., Luinge H.J., Baten C.T.M., Veltink P.H. (2005). Compensation of Magnetic Disturbances Improves Inertial and Magnetic Sensing of Human Body Segment Orientation. IEEE Trans. Neural Syst. Rehabil. Eng..

[36] Luinge H.J., Veltink P.H., Baten C.T.M. (2007). Ambulatory measurement of arm orientation. J. Biomech..

[37] Roetenberg D., Luinge H., Slycke P. (2013). Xsens MVN: Full 6DOF Human Motion Tracking Using Miniature Inertial Sensors.

[38] Gallagher S., Sesek R.F., Schall M.C., Huangfu R. (2017). Development and validation of an easy-to-use risk assessment tool for cumulative low back loading: The Lifting Fatigue Failure Tool (LiFFT). Appl. Ergon..

[39] Gallagher S., Schall M.C., Sesek R.F., Huangfu R. (2019). Assessment of Job Rotation Effects for Lifting Jobs Using Fatigue Failure Analysis. Adv. Intell. Syst. Comput..

[40] Schall M.C., Fethke N.B., Chen H. (2016). Working postures and physical activity among registered nurses. Appl. Ergon..

[41] Granzow R.F., Schall M.C., Smidt M.F., Chen H., Fethke N.B., Huangfu R. (2018). Characterizing exposure to physical risk factors among reforestation hand planters in the Southeastern United States. Appl. Ergon..

[42] Marras W.S., Lavender S.A., Ferguson S.A., Splittstoesser R.E., Yang G. (2010). Quantitative biomechanical workplace exposure measures: Distribution centers. J. Electromyogr. Kinesiol..

[43] Davis K.G., Orta Anés L. (2014). Potential of adjustable height carts in reducing the risk of low back injury in grocery stockers. Appl. Ergon..

[44] Shojaei I., Vazirian M., Croft E., Nussbaum M.A., Bazrgari B. (2016). Age related differences in mechanical demands imposed on the lower back by manual material handling tasks. J. Biomech..

